# A model for estimating pathogen variability in shellfish and predicting minimum depuration times

**DOI:** 10.1371/journal.pone.0193865

**Published:** 2018-03-07

**Authors:** Paul McMenemy, Adam Kleczkowski, David N. Lees, James Lowther, Nick Taylor

**Affiliations:** 1 Computing Science and Mathematics, Faculty of Natural Sciences, University of Stirling, United Kingdom; 2 Food Safety Group, CEFAS, Weymouth, United Kingdom; 3 Epidemiology Team, CEFAS, Weymouth, United Kingdom; Bigelow Laboratory for Ocean Sciences, UNITED STATES

## Abstract

Norovirus is a major cause of viral gastroenteritis, with shellfish consumption being identified as one potential norovirus entry point into the human population. Minimising shellfish norovirus levels is therefore important for both the consumer’s protection and the shellfish industry’s reputation. One method used to reduce microbiological risks in shellfish is depuration; however, this process also presents additional costs to industry. Providing a mechanism to estimate norovirus levels during depuration would therefore be useful to stakeholders. This paper presents a mathematical model of the depuration process and its impact on norovirus levels found in shellfish. Two fundamental stages of norovirus depuration are considered: (*i*) the initial distribution of norovirus loads within a shellfish population and (*ii*) the way in which the initial norovirus loads evolve during depuration. Realistic assumptions are made about the dynamics of norovirus during depuration, and mathematical descriptions of both stages are derived and combined into a single model. Parameters to describe the depuration effect and norovirus load values are derived from existing norovirus data obtained from U.K. harvest sites. However, obtaining population estimates of norovirus variability is time-consuming and expensive; this model addresses the issue by assuming a ‘worst case scenario’ for variability of pathogens, which is independent of mean pathogen levels. The model is then used to predict minimum depuration times required to achieve norovirus levels which fall within possible risk management levels, as well as predictions of minimum depuration times for other water-borne pathogens found in shellfish. Times for *Escherichia coli* predicted by the model all fall within the minimum 42 hours required for class B harvest sites, whereas minimum depuration times for norovirus and FRNA+ bacteriophage are substantially longer. Thus this study provides relevant information and tools to assist norovirus risk managers with future control strategies.

## Introduction

Norovirus (NoV) is one of the dominant causes of global food-borne illness. In 2011 in the United States of America alone, an estimated 58% of 9.4 million cases of food-borne illness were attributed to norovirus [1]. A global increased occurence of NoV has been reported [2], with children under 5 years old in developing countries deemed to be particularly vulnerable to the effects of acute gastroenteritis [3]. One pathway identified for norovirus to pass into the human population is the consumption of bivalve shellfish [4, 5]. Shellfish filter-feed nutrients from their surrounding waters which, in addition to feeding, can concentrate contaminants and infectious agents often associated with faecal contamination into their digestive system [4, 6–9]. The potential exists for transmission of such agents into the human population if the shellfish are consumed while they still contain such pathogens. This is of special concern when shellfish are eaten raw, which is commonly the case for oysters such as the Pacific cupped oyster (recently reclassified as *Magallana gigas* from *Crassostrea gigas* [10]) and the American cupped oyster (*Crassostrea virginica*) [11].

To protect against the accumulation of pathogens in shellfish, farms should ideally be situated in waters with low pollution levels. However, due to socio-geographic reasons, this is not always possible as many farms are located close to population centres [12–14]. There have been recent increases in the volumes of most fish species farmed via aquaculture processes; however, global mollusc production has remained relatively constant since 1990. Oyster production has exhibited a slight downward trend over the same time period [15], potentially due to NoV outbreaks being linked to oyster consumption which has been more frequently indicated in recent times [16].

In most industrialised countries, legislation has been put in place to minimise levels of faecal contamination found in shellfish, and thus reduce health risks to consumers. European levels of faecal contaminants in shellfish are legislated for by EU Regulation (EC) No 853/2004, which states that “live bivalve molluscs must come from: (a) a class A production area; (b) a relaying area; (c) a purification centre…” [17]. Shellfish harvest sites are classified as A, B, or C based on levels of the faecal indicator organism *Escherichia coli* (*E. coli*) detected in the shellfish [17]. A relaying area is a class A or B rated site where shellfish harvested from class B and C waters are relocated for a time sufficient to reduce faecal contamination to acceptable levels based on *E. coli* counts. Class A waters can be limited in availability, and so in many instances an alternative to farming or relaying in these areas is required [17, 18].

Depuration is the most common alternative, a process which resubmerges harvested shellfish in tanks containing clean water, where they remain for a period of time sufficient for the animals to excrete any microbiological contaminants they may contain [8, 11, 19–21]. Currently, in the U.K., a minimum depuration period of 42 hours exists for shellfish harvested from a class B area [7, 18, 22, 23], which has been shown to be sufficient to reduce *E. coli* counts to less than 230 *E. coli*/100g.

Although *E. coli* is a reliable indicator of faecal pollution, it is a poor indicator of viral and chemical contamination in shellfish [6, 24, 25], and viral contaminants such as NoV are not currently directly controlled under any national or E.U. legislation [26]. At present, NoV levels within shellfish are reduced only by methods put in place to mitigate other contaminants, despite posing a potential risk to consumer health [4, 27, 28]. Although several countries have conducted monitoring for viral contamination, thus far no producer countries have implemented legislative standards [29–31].

The effectiveness of depuration in reducing microbiological loading relies on a number of criteria: water temperature, salinity, oxygenation and flow rates, as well as hygiene controls implemented when cleaning tanks [20, 22, 23, 32]. Water temperature and salinity should closely match the harvest location’s conditions to minimise stress to the shellfish; the oxygen levels in the water should be sufficient for the density of shellfish held in the tank to allow normal metabolic activity in the animals; and the cleaning processes between depuration cycles must ensure that any excreted contaminants in the tank are removed before a new shellfish batch is processed. In addition to its importance in terms of animal welfare, the water temperature is also an important factor in removing NoV and other contaminants from oysters. Studies by Neish (2013) and Doré (2010) indicate that higher water temperatures can increase the rate of NoV excretion [33, 34]. However, there is also evidence in the literature that depuration has only a limited impact on NoV loads [20, 34, 35], with the 42 hour minimum depuration time to remove *E. coli* [4, 7, 34] having only a limited effect on NoV loads in comparison to its effect on *E. coli* and other viral contaminants [36].

Since depuration can incur significant costs to the shellfish industry [7, 20, 34], minimising any costs while at the same time minimising NoV levels in shellfish would be beneficial to both the industry and the consumer. Thus an increased understanding of the dynamics of NoV loads during depuration is crucial in order to determine the time required to reduce NoV to safe levels whilst minimising costs.

Using mathematical approaches, this paper aims to model the depuration process to determine how NoV (as well as other water-borne pathogens) levels in shellfish batches change over time spent in depuration. This is based on the initial pathogen values; the way in which the pathogen loads evolve over time is also incorporated into the model. In particular the study considers the following questions to construct and test the model: *(i)* What is the distribution of NoV loads at the beginning of the depuration and how does this change over time? *(ii)* What is the probability that the NoV load in a randomly sampled shellfish exceeds a defined threshold value? *(iii)* What duration of depuration is required to reduce the potential risk of an shellfish containing NoV loads above such a threshold being sold for consumption? *(iv)* Does the model apply to other water-borne pathogens, such as *E. coli*? *(v)* Do depuration times modelled for *E. coli* meet the minimum 42 hours depuration criterion?

## Model

### Pre-depuration pathogen distribution

We begin by defining a probability density function (p.d.f.) for NoV loads within a shellfish population, *X*_0_ = *x*_0_, at the pre-depuration time *t* = 0 as *P*(*x*_0_). We assume that NoV loads in such a population are well-approximated by a log-normal distribution, as many other water-borne pathogens have been previously shown to be well described by log-normal behaviour [20, 37–40]. Thus *P*(*x*_0_) is described by $LN(\mu_{0},\sigma_{0}^{2})$, where *μ*_0_ is the mean of the log-values of NoV copies per gram (cpg) per shellfish, and *σ*_0_ is the standard deviation of the log-values of NoV cpg per shellfish. It follows that the arithmetic mean (or expected value) and standard deviation of *P*(*x*_0_) are defined as ${\bar{x}}_{0}=E\left(x_{0}\right)=\text{exp}\left\{\mu_{0}+\sigma_{0}^{2}/2\right\}$ and $SD\left(x_{0}\right)=\sqrt{\text{exp}\{\sigma_{0}^{2}\}-1}\;\text{exp}\left\{\mu_{0}+\sigma_{0}^{2}/2\right\}$ respectively.

### Depuration pathogen distribution

A probability distribution, *P*(*x*_*t*_), that describes the distribution of NoV during depuration (*x*_*t*_) can be derived by assuming that *x*_0_ is related to *x*_*t*_ by some continuous function *x*_*t*_ = *f*(*x*_0_; *t*). Previous studies, such as those by Polo *et al* [41, 42], have demonstrated that pathogen decay due to depuration is well-approximated by a exponential term, and so we assume an exponential decay of NoV loads per shellfish due to the depuration process, in line with the current literature [32, 41–44]. Thus *x*_*t*_ = *x*_0_ exp{−*bt*}, where *b* is the decay rate specific to the efficacy of the depuration process applied. It can be shown that *P*(*x*_*t*_) is defined by $LN(\mu_{0}-bt,\sigma_{0}^{2})$, or

$$\begin{array}{c} P\left(x_{t}\right)=\frac{1}{\sqrt{2\pi }\sigma_{0}\;x_{t}}\text{exp}\{\frac{-{\left(\text{ln}\left(x_{t}\right)+bt-\mu_{0}\right)}^{2}}{2\sigma_{0}^{2}}\}\;. \end{array}$$ (1)

The mean value of *P*(*x*_*t*_) is ${\bar{x}}_{t}=\text{exp}\left\{\mu_{0}-bt+\sigma_{0}^{2}/2\right\}$, with the standard deviation of the depuration distribution also decaying at the same rate: SD(*x*_*t*_) = SD(*x*_0_) exp{−*bt*}. From these results, it follows that *σ*_*t*_ = *σ*_0_ and that *μ*_*t*_ = *μ*_0_ − *bt*.

### Tail of the distribution

Eq 1 describes a positively skewed p.d.f. where the *tail* of the p.d.f. is defined as including values of *x*_*t*_ greater than Ψ, a measure of pathogen concentration per shellfish below which a high level of food safety is provided to the consumer (see S1 Fig). The area within the tail of the p.d.f., *P*(*x*_*t*_ > Ψ), is of particular interest as it quantifies the risk of marketing ‘non-compliant’ shellfish i.e. shellfish containing pathogen loads exceeding Ψ. At time *t*, the probability of a randomly selected shellfish having a pathogen level greater than Ψ is given by

$$\begin{array}{c} P\left(x_{t}>\Psi \right)=\int_{\Psi }^{\infty }\frac{1}{\sqrt{2\pi }\sigma_{0}\;x_{t}}\text{exp}\{\frac{-{\left(\text{ln}\left(x_{t}\right)+bt-\mu_{0}\right)}^{2}}{2\sigma_{0}^{2}}\}\;\;dx_{t}=1-\varphi \;\text{.} \end{array}$$ (2)

Mathematically, some remnant of the tail will never fall below the value of Ψ, as the value of *P*(*x*_*t*_ > Ψ) will decay to zero only as *t* → ∞. For the log-normal distribution in Eq 1, there is always a non-zero probability *P*(*x*_*t*_) > Ψ, i.e. there is always a probability that the NoV load in an randomly selected shellfish may exceed the threshold level Ψ. Public health authorities would require that this probability value be very small. Therefore we define an additional variable in the model as an *assurance level*, *φ*, which is the probability that a randomly sampled shellfish at time *t* will have a NoV load below the threshold value, Ψ. Thus the tail of Eq 2 is also quantified by 1 − *φ*, and so to minimise any food safety implications, the value of *φ* ≈ 1. Both of these values, Ψ and *φ*, could be defined by public health authorities [26]. All parameters used in Eq 2 are defined and described in Table 1.

**Table 1:** Parameter definitions.

Parameter	Definition	Description	Units
*t*	depuration duration	Length of depuration time modelled	hr
*μ*_*t*_, *σ*_*t*_	lognormal distribution parameters	Location and variability parameters of the log-transformed distribution at time *t*	cpg
Ψ	pathogen threshold limit	Pathogen load value above which an individual shellfish may present a food safety risk	cpg
*φ*	pathogen assurance level	Proportion of shellfish population in depuration which must have pathogen loads less than Ψ at the end of depuration	cpg
*b*	depuration decay rate	Reduction rate of pathogens within individual shellfish due to depuration process	hr^−1^
${\bar{x}}_{0}$	initial mean pathogen load	Arithmetic average of pathogen distribution at post-harvest/pre-depuration *t* = 0	cpg

### Minimum depuration time

Solving Eq 2 for time *t*, we obtain a value for the minimum depuration time (MDT), *t* = *T*(*μ*_0_, *σ*_0_), the time that is required for the shellfish to be depurated to satisfy the terms of *P*(*x*_*t*_ > Ψ) = 1 − *φ*. This MDT is given by

$$\begin{array}{c} T\left(\mu_{0},\sigma_{0}\right)=b^{-1}[\sqrt{2}\sigma_{0}\;{\text{erf}}^{-1}(2\varphi -1)-\text{ln}\left(\Psi \right)+\mu_{0}]\;, \end{array}$$ (3)

where erf^−1^ is the inverse error function.

Eq 3 above requires estimates of both parameters *μ*_0_ and *σ*_0_; however, the current standard assay for NoV detection utilises homogenates of 10 oysters [45], and so only provides an arithmetic mean NoV cpg (${\bar{x}}_{0}$) of the 10 oysters sampled by the assay. In relation to the lognormal distribution, the arithmetic mean is related to *μ*_0_ and *σ*_0_ by $\mu_{0}=\text{ln}\left({\bar{x}}_{0}\right)-\sigma_{0}^{2}/2$ [46]. Obtaining estimates of *σ*_0_ would require many test iterations to be performed on individual oysters, which is both time-consuming and costly [34]. We can provide a ‘worst case scenario’ for variability using our model, i.e. provide an upper limit minimum depuration time. Eq 3 and $\mu_{0}=\text{ln}\left({\bar{x}}_{0}\right)-\sigma_{0}^{2}/2$ are combined and rearranged to present *T*(*μ*_0_, *σ*_0_) in terms of only *σ*_0_:

$$\begin{array}{c} T\left(\sigma_{0}\right)=b^{-1}[-\frac{1}{2}\sigma_{0}^{2}+\sqrt{2}{\text{erf}}^{-1}(2\varphi -1)\sigma_{0}+\text{ln}(\frac{{\bar{x}}_{0}}{\Psi })]\;. \end{array}$$ (4)

Eq 4 is in concave quadratic form with respect to *σ*_0_, and so is maximised by

$$\begin{array}{c} \sigma_{0}=\sqrt{2}{\text{erf}}^{-1}\left(2\varphi -1\right)\;, \end{array}$$ (5)

Thus the selection of the assurance level *φ* also establishes the maximum value of *σ*_0_ at a ‘worst case scenario’ level with respect to minimum depuration time. This worst case variability (WCV) is a consequence of the assumption of lognormality, which is truncated at *x*_*t*_ = 0 yet has no upper limit. As variability (*σ*_0_) is increased, the distribution shape becomes increasingly bunched at lower values of *x*_*t*_, while simultaneously increasing the density of the distribution’s tail. The WCV is therefore dependent on the proportion of the area under the curve (*φ*) which is required to fall below the NoV threshold value, Ψ. This process is fully described by Eq 4, but can be redefined in terms excluding *σ*_0_ by substituting in Eq 5:

$$\begin{array}{c} T_{\text{WCV}}=b^{-1}\left[{\left({\text{erf}}^{-1}\left(2\varphi -1\right)\right)}^{2}+\text{ln}\left(\frac{{\bar{x}}_{0}}{\Psi }\right)\right], \end{array}$$ (6)

where *T*_WCV_ is the minimum depuration time required to satisfy the constraints of the parameters *φ* and Ψ, while assuming a WCV of the pathogen across the shellfish population.

Assuming that the parameters which control the food safety constraints of our model (*φ* and Ψ) would be fixed by legislation in the future, then only the depuration efficacy of the process being used (*b*) and the estimate of the average NoV load for the shellfish population (${\bar{x}}_{0}$) are required to calculate MDTs.

The expected value of Eq 1 was previously defined as $E\left(x_{t}\right)=\text{exp}\left\{\mu_{0}-bt+\sigma_{0}^{2}/2\right\}$. This, once *T*_WCV_ has been achieved, can be restated as

$$\begin{array}{c} E\left(x_{T_{WCV}}\right)=\Psi \text{exp}\left\{-{\left({\text{erf}}^{-1}\left(2\varphi -1\right)\right)}^{2}\right\}. \end{array}$$ (7)

This describes the arithmetic mean of the shellfish population at the point of MDT, subject to the control parameters Ψ and *φ*. Note that Eq 7 is calculated solely from these same control parameters.

## Results

### Pre-depuration parameterisation

We use a combination of data from the literature to derive parameters for NoV to use with the model. Lowther *et al* analysed NoV loads in oysters from 39 U.K. harvest sites in 2010–11, with samples collected each month over the two year period [47]. The 39 sites were comprised of 6 class A, 31 class B and 2 class C sites from around mainland Britain. Class A sites exhibited low NoV loads; by legislation, class C sites must use relaying rather than depuration to reduce any contaminants, so only the class B data were analysed to obtain estimates of arithmetic mean values (${\bar{x}}_{0}$) of NoV. The observations recorded in winter months exhibited higher NoV concentrations, with Class B Jan 2010 average ${\bar{x}}_{0}=1062$ NoV cpg, and similarly with Jan 2011 ${\bar{x}}_{0}=1064$ NoV cpg.

**Fig 1:**
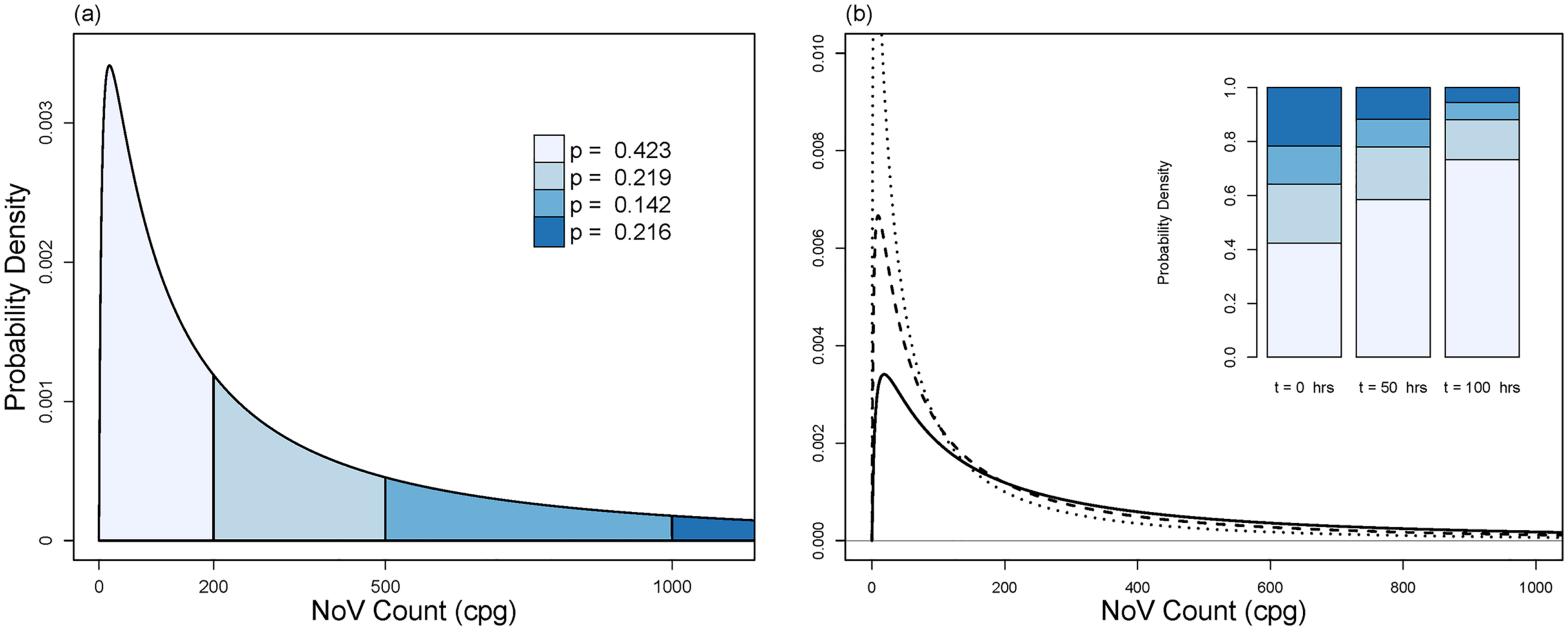
Pre-depuration (a) and during depuration (b) probability distributions, with ${\bar{x}}_{0}=1064$ NoV cpg, and *σ*_0_ = 1.645. This variability corresponds to the worst case scenario where *φ* = 95% (Eq 5). Fig (a) splits the distribution’s area into four sections, and states probabilities for each section. Fig (b) shows probability distributions at *t* = 0 hrs (**——**), *t* = 50 hrs (**– – –**), *t* = 100 hrs (⋯) induced by depuration decay rate *b* = 0.01339. Note the different vertical axis scales. Inset bar plot shows the respective changes in section probabilities for each time point corresponding to domain values in Fig 1(a).

The pre-depuration distribution, *P*(*x*_0_), of Class B Jan 2011 sampled oysters (${\bar{x}}_{0}=1064$ NoV cpg) is shown in Fig 1(a), assuming a WCV of *σ*_0_ = 1.645, equivalent to an assurance level of *φ* = 95% (cf. Eq 5).

The distribution was used to calculate the probability that a randomly sampled oyster’s NoV load fell within a particular range. It was calculated that there was only a 42.3% likelihood that a randomly sampled oyster would have a NoV load below Ψ = 200 NoV cpg. Also, there was a 21.6% probability that an oyster before depuration would have a NoV cpg load greater than 1000 cpg using this WCV approach.

### NoV dynamics during depuration

#### Depuration decay rate parameterisation

Doré *et al* [33] carried out a 2010 survey of an Irish farm which had recognised they were selling oysters with greater than expected NoV levels. The farm voluntarily applied additional NoV mitigation methods to reduce any potential risk to their consumers. In the published report, the authors provide pre-depuration and during depuration data on NoV levels detected in oysters using a quantitative PCR assay (see Table 2).

**Table 2:** Depuration decay rates for NoV derived from data in Doré *et al* [33].

	Pre-Dep. (${\bar{x}}_{0}$)	During Dep. (${\bar{x}}_{96}$)	Post-Dep. (${\bar{x}}_{144}$)
NoV cpg	492	136	99 (max.)
Total duration (hrs)	0	96	144
Decay rate, *b*	—	0.01339	0.01113

The exponential decay of pathogen loads due to depuration was previously defined by *x*_*t*_ = *x*_0_ exp{−*bt*}. Using this equation and inputting the values for ${\bar{x}}_{0}$, ${\bar{x}}_{96}$ and ${\bar{x}}_{144}$ shown in Table 2, depuration decay rate *b* can be established for 0–96 hours (*b* = 0.01339) and 0–144 hours (*b* = 0.01113).

The same paper also states “Since 19 March 2010 more than 50,000 oysters have been placed on the market and no reports of illness have been received. NoV levels in these batches were less than 200 viral genome copies per g” [33]. This provides a parameter value estimate of Ψ = 200 cpg specifically for NoV. Note that this value is being used for the purpose of this study and is not a recommendation for policy purposes.

#### Distribution dynamics during depuration

The dynamics of the depuration model are analysed using the Class B Jan 2011 data and applying the estimated depuration decay rate derived from Doré (2010) (*b* = 0.01339) [33] for a reasonable duration (0–100 hours). The exponential decay of NoV loads in individual oysters determines the dynamics of the population and is characterised by *P*(*x*_*t*_) (Fig 1(b)). The peak of the highly-skewed distribution moves towards lower values of *x*_*t*_, creating a distribution with area that is increasingly located close to zero cpg. With the NoV threshold limit set at Ψ = 200 cpg, the model shows 42.3% of oysters would be below this NoV load level at *t* = 0. After *t* = 50 hours, 58.4% are below Ψ, while 73.2% are below the threshold after 100 hours of depuration.

### NoV minimum depuration times

MDTs are calculated for Class B Jan 2011 data (${\bar{x}}_{0}=1064$ cpg) using Eq 6 and applying Ψ = 200 NoV cpg, and varying values of *φ* = 90%, 95% and 99% assurance levels. MDTs of 186, 226, & 327 hrs were required to satisfy the respective *φ* = 90%, 95% and 99% assurance levels.

In practice, the rate of depuration could vary quite substantially; we therefore carried out sensitivity analysis with respect to *b*. The depuration rate obtained from Doré (2010) [33] was used as a baseline, and −25%, −10%, +10%, +25%, +50%, +100% depuration rates were used to calculate MDTs, applying the Class B Jan 2011 NoV average (${\bar{x}}_{0}=1064$ NoV cpg). Results for MDTs for each parameter pair (*b*, *φ*) are shown in Table 3. Increasing depuration efficacy by 10% provided a 9.09% decrease in depuration time; increasing depuration efficacy by 25% decreased time required by 20.01%; depuration efficacy increased by 50% decreased time required by 33.30%; doubling depuration efficacy halved the required depuration time. These results are a consequence of Eq 6, and show that a change in MDTs of a factor of *ρ*/(1 + *ρ*) occurs if depuration efficacy is altered by a proportion *ρ*. It is worth noting that if depuration efficacy falls by (e.g.) 25%, then minimum depuration times would need to be increased by 33.33%.

**Table 3:** Minimum depuration times (*T*_WCV_) for varying decay rates and NoV assurance levels. Simulated NoV test results of 10-oyster homogenates, which had undergone depuration using each parameter set {*b*, *φ*, *T*_WCV_} are shown. Times are in hours.

Dep. Decay Rate (hr^−1^)	*φ* = 90%	*φ* = 95%	*φ* = 99%
*b* − 25%	248.2	301.1	435.9
*b* − 10%	206.8	251.0	363.2
*b* = 0.01339	186.2	225.9	326.9
*b* + 10%	169.2	205.3	297.2
*b* + 25%	148.9	180.7	261.5
*b* + 50%	124.1	150.6	217.9
*b* + 100%	93.1	112.9	163.5
Pass Rate	96%	98%	99%

The model constructed in this paper can be used to simulate current NoV test protocols, and so determine whether post-depuration testing of shellfish populations that had undergone depuration for time *t* ≥ *T*_WCV_ would pass current testing practices. This was accomplished by constructing probability density functions that represent the distribution of pathogens within the population for each time *t* = *T*_WCV_ shown in Table 3, where each *T*_WCV_ has a unique parameter set {*b*, *φ*, *T*_WCV_}. Scale parameters (*μ*_*t*_) for each distribution were obtained from *μ*_*t*_ = *μ*_0_ − *bT*_WCV_, and variability was set at worst case levels (Eq 5). Ten random variates were selected from each distribution and their mean was calculated, with each mean simulating a 10-shellfish homogenate. This mean would be deemed a test ‘pass’ if it’s value was less than 200 NoV cpg, and a test ‘fail’ if otherwise. Ten thousand iterations of each parameter set were run using the model, with the proportion of test passes shown in Table 3.

Using assurance levels of *φ* = 90%, 95% and 99%, pass rates of approximately 96%, 98% and 99% were achieved for the minimum depuration times shown for *φ* = 90%, 95%, 99% respectively. Further analysis of these pass rates is presented in S2 Fig

### E. coli and FRNA+ minimum depuration times

As previously stated, E. coli is used to classify water quality as well as determine the minimum 42 hour depuration period required for shellfish harvested from class B waters. However, E. coli has been shown to be a poor indicator of the presence of viral contaminants in water; F-positive RNA bacteriophage (FRNA+) has been suggested as an improved correlate for the presence of viral contaminants [24, 48]. Analysis of MDTs derived from parameters that describe the distribution and depuration rates of these contaminants would be of significance in validating the model, as the literature shows that, in all cases, E. coli levels are reduced to below the limit of detection (LOD) by 42 hours of depuration [7, 18, 22, 23].

Oyster depuration decay rates for E. coli and FRNA+ were derived from Doré et al (1995) [6], who provided durations for pre-depuration pathogen levels to fall by 90% (designated there as T90). For E. coli, T90 was between 6–7 hours, with FRNA+ between 55–60 hours. Applying our assumption of exponential decay, these T90s translate into decay rates of E. coli: 0.38376–0.32894; FRNA+: 0.04187–0.03838. Again adopting a worst case approach, the lower values of these are used to parameterise decay rates for both pathogens to calculate the *T*_WCV_s (Table 4).

**Table 4:** Sensitivity of minimum depuration times (*T*_WCV_) to parameters ${\bar{x}}_{0}$, Ψ for common water-borne pathogens found in shellfish.

			*b*	${\bar{x}}_{0}$	Ψ	*T*_WCV_	*T*_WCV_	*T*_WCV_
			(hr^−1^)	(c/100g)	(c/100g)	*φ* = 90%	*φ* = 95%	*φ* = 99%
NoV^a^	low ${\bar{x}}_{0}$	low Ψ	0.01339	2 780^b^	300^c^	227.6	267.3	368.4
		high Ψ			2 000	85.9	125.6	226.7
	high ${\bar{x}}_{0}$	low Ψ		10 640	300^c^	327.8	367.5	468.6
		high Ψ			2 000	186.2	225.9	326.9
*E. coli*	low ${\bar{x}}_{0}$	low Ψ	0.32894	180 000^c^	20	30.2	31.8	35.9
		high Ψ			230	22.8	24.4	28.5
	high ${\bar{x}}_{0}$	low Ψ		400 000^c^	20	32.6	34.2	38.3
		high Ψ			230	25.2	26.8	30.9
FRNA+	low ${\bar{x}}_{0}$	low Ψ	0.03838	10 471^d^	30^f^	174.0	187.8	223.1
		high Ψ			300	114.0	127.8	163.1
	high ${\bar{x}}_{0}$	low Ψ		80 000^e^	30^f^	226.9	240.8	276.0
		high Ψ			300	166.9	180.8	216.0

^a^ Note the scaling up to units of c/100g

^b^ [33]

^c^ [26]

^d^ [48]

^e^ [6]

^f^ [48, 49]

The same study provided initial contamination levels in oysters ranging from 180 000 to 400 000 E.coli organisms, and 70 000 to 80 000 F^+^ bacteriophage per 100g [6]. Again, we select the worst case from these values, this time using the upper values of the ranges to parameterise ${\bar{x}}_{0}$ for E. coli and FRNA+. However, these values are from shellfish exposed to secondary-treated effluent at a distance of 800 metres for 3 weeks and so may reflect artificially high contamination levels. Flannery et al (2013) provides mean FRNA+ loads in oysters using RT-qPCR as 10471 c/100g, and 1380 PFU/100g using plaque assay [48]. Again we will apply the greater, worst case values to our model.

Parameterising the load limit (Ψ) for E. coli and FRNA+ is based on the LOD for both pathogens, with E. coli’s LOD stated as 20 E.coli per 100g [20], and for FRNA+ as 30 PFU [plaque forming units] 100 g^−1^ using plaque assay [48, 49].

Minimum depuration times for NoV, E. coli and FRNA+ are shown in Table 4 using these parameters. The MDTs calculated for E. coli range from 25.2–30.9 hrs with Ψ = 230 c/100g; these times are well below the minimum duration of 42 hours depuration from class B sites. Applying a more stringent value of Ψ = 20 c/100g (the LOD for E. coli) still results in all MDTs calculated as being less than the minimum 42 hrs. We have applied a mean value of 400 000 copies/ 100g for E. coli: a value which would result in a “Prohibited”classification and immediate closure of the site if recorded for a single shellfish. Coupling this with our use of a worst case variability provides a strong validation for the minimum time of 42 hours depuration from class B harvest sites, as well as a tentative validation of this model.

The MDTs calculated for FRNA+ (low ${\bar{x}}_{0}$) bacteriophage are more in line with the MDTs for NoV, however they exhibit a much narrower range across the values of assurance levels applied.

## Discussion and conclusions

NoV is a significant cause of gastroenteritis globally [1, 3], and the consumption of oysters is linked to outbreaks [5, 27]. For products placed live on the market, depuration is the principle means employed to reduce levels of potentially harmful agents in shellfish [35]. Though the minimum depuration times for faecal indicator organisms such as coliform bacteria are well established [18, 22], little data is currently available to inform these times for NoV. This study provides a mathematical framework that could be used to help determine the minimum depuration times required to reduce NoV levels to below a desired threshold. This model is based on the well documented assumptions that NoV is log-normally distributed throughout a population of oysters [20, 37–40] and that pathogen load decay during depuration is exponential [32, 42–44]. The model requires the input of four parameters: i) the initial average NoV load, ii) the depuration efficacy, iii) the desired assurance level and iv) the required NoV threshold. Based on these inputs the model provides an estimate of the minimum depuration time required to reduce norovirus levels below the desired threshold. This, in conjunction with the other parameters, can also be used to determine the probability of a batch of oysters testing below the detection threshold after depuration. A protocol for determining minimum depuration times using the model is as follows:

1. Measure ${\bar{x}}_{0}$ of oyster batch’s harvest site;
2. Determine characteristic efficacy of overall depuration process, *b*^−1^;
3. Fix value of NoV load threshold, Ψ;
4. Select NoV assurance level, *φ*;
5. Apply these parameter values to the model to obtain recommended depuration period, *T*_WCV_ (cf. Eq 6).

Steps 1–3 are anticipated to be assessed or fixed by public health authorities. The NoV assurance level parameter *φ* may not be fixed (e.g. by legislation); however, applying larger values of this parameter in the model would provide increased confidence to both depurators and consumers, as this would require a greater proportion of the population’s pathogen load to be less than Ψ, and so would extend predicted MDTs. This would ensure that oysters passing into the supply chain would have a diminished probability of containing significant NoV levels.

The initial NoV load is determined using the international standard for quantification of NoV in foods [45] prior to depuration. This test provides a NoV load for a pooled sample of ten oysters, assumed to be the arithmetic mean of the the loads in the individual oysters within the population of ten. However this provides no information on variability within the population which is required in the calculation of depuration times. However, a worst case level of variability can be determined in the absence of this data. This worst case variability increases with the desired assurance level (see Eq 5), as does the depuration time required. Further work would be required to accommodate terms describing pathogen replication within shellfish during depuration, and are not included as NoV has previously been reported as being only carried, and not replicated, whilst within shellfish [4]. As real data becomes available for variability in NoV between oysters, this can be substituted for the worst case variance, which will result in a reduction in the predicted depuration times. Seasonal variation of pathogen levels (as observed in the NoV data for Jan 2010/11 in comparison with all summer months examined by Lowther et al [47]) could be accounted for by the application of appropriately gauged values of ${\bar{x}}_{0}$.

Little data is currently available regarding the depuration efficacy of different systems for the removal of NoV. For illustrative purposes this study looks at the scenarios resulting from estimates obtained from Doré et al (2010) [33], but clearly there needs to be substantial research to determine accurate depuration efficacies for NoV before sensible predictions regarding depuration times can be made. This (or any other) model requires extensive time series data collected from a controlled environment to derive accurate depuration decay rates, and is outside the remit of this current work. However, regardless of the value used, it is possible to calculate the relationship between improvements in depuration efficacy and depuration time, which approximately halves as efficacy doubles.

The model’s main assumptions of lognormality (used to describe the distribution of pathogens across a shellfish population) and exponential decay of pathogen levels during depuration are approximations of a reality which is discrete. Consideration of discretized models, as well as non-linear terms describing the pathogen decay rate, may provide more realistic descriptions of depuration dynamics and will be considered in future work. Further work could also examine imposing a maximum pathogen load that can be carried by a shellfish. This would reflect a realistic upper limit on the amount of pathogen that can be carried (per unit), and which may remove the need to apply the assurance level (*φ*).

The assumption of constant exponential decay across the population made in this model does not take into account any variation in pathogen decay rates between individual shellfish. In reality, these rates would vary across the population; however, obtaining reliable parameters to inform any such variation would require significant experimental data and again is outside the scope of this work.

The assurance level (set by the regulator) determines the desired proportion of oysters in the population with NoV levels below the set threshold after depuration. This is important as, in addition to providing a confidence level associated with the safety of a batch depurated oysters, it is also directly linked to the probability that a sample of ten oysters will return a value below the threshold after depuration. Though increasing the assurance level also increases the required depuration time, it will also reduce the probability that a batch of oysters will fail testing, thus allowing risk managers to evaluate the tradeoffs between depuration times and an acceptable failure rate.

The final parameter is the pathogen threshold limit, Ψ, which is likely to be set by an appropriate regulating body. The ratio between the values of the initial average pathogen level (${\bar{x}}_{0}$) and the threshold limit value greatly impacts the length of depuration required (see Eq 6). Though this study uses a value of 200 NoV cpg, this is purely for illustrative purposes and not a suggestion for such a limit, which would require a detailed understanding of the health risk posed by different levels of NoV.

Parameters describing the depuration decay rate, pathogen threshold limit and pre-depuration mean value for E. coli and FRNA+ have been identified from scientific literature. These have been applied to the model and the resultant minimum depuration times obtained for E. coli are all within the 42 hours of depuration currently legislated for class B harvests. Even when applying the worst case variability to E. coli levels that massively exceed those expected to be found in class B shellfish, the minimum depuration times were still less than 42 hours. This not only provides further confidence in the legislated minimum depuration time, but also provides some level of validation to the model introduced here. Current E.U. regulations only stipulate pathogen limits for E. coli and coagulase-positive staphylococci for shellfish production [50]; this model can be used to provide minimum depuration times for other microbiological and viral pathogens beyond those already legislated for such as NoV and FRNA+ bacteriophage.

In conclusion, depuration is one of the tools through which shellfish industries aim to reduce NoV to an acceptable level. This study arose from a desire to provide a useful framework to help industry and regulators understand the relationship between possible future, desired NoV levels and required depuration times. In doing so this also provides a tool with which to determine by how much depuration efficacies may need to improve to reduce depuration times to levels deemed economically and logistically feasible by industry. Having the ability to determine the depuration times required to bring NoV loads to below threshold levels should be provide a useful tool to both producers and risk managers.

## Supporting information

S1 Fig Shapes of *n*-summed distributions *P*(*x*_*t*, *n*_). Distributions shown with sample sizes *n* ∈ {10, 30, 50, 100}.
(PDF)

S2 Fig Results of test simulations of *n*-sized homogenates. Results shown are based upon applying an assurance level *φ* = 95% (**– – –** line).
(PDF)

S3 FigPlot of pathogen variability (*σ*_0_) versus minimum depuration time (*T*(*σ*_0_)).Threshold limit Ψ = 200 NoV cpg, NoV assurance level *φ* = 95%, and initial mean NoV load ${\bar{x}}_{0}=1064$ cpg.(PDF)
