# Supplementary material for: A model for estimating pathogen variability in shellfish and predicting minimum depuration times
Source: PLoS One. 2018 Mar 7;13(3):e0193865. doi: 10.1371/journal.pone.0193865 (PMC5841822; doi:10.1371/journal.pone.0193865)
Supplement: S1 Fig — Distributions shown with sample sizes n ∈ {10, 30, 50, 100}. (PDF) [file pone.0193865.s001.pdf]

S1 Fig. Table 3 (Minimum Depuration Times) in the main text shows that the test pass rates for 10-oyster homogenates are 96%, 98%, 99% when $\varphi = 90\%,\ 95\%,\ 99\%$ respectively. This is a consequence of both the asymmetric shape of a lognormal distribution and the Central Limit Theorem (CLT). CLT states that as n independent identically distributed (i.i.d.) random variables are added, then their properly normalized sum tends toward a normal distribution. This holds even when the the original i.i.d. variables are not themselves normally distributed. This behaviour has implications for the homogenate sample size used by PCR to detect NoV (and other lognormally distributed water-borne pathogens). Current PCR homogenates are comprised of the digestive tracts of 10 oysters [1], which sums the NoV loads of the 10 shellfish. This results in a summed distribution that transitions into a less asymmetric distribution, with the middle of the 10-summed distribution increasing as a result of the summation when compared with the distribution of the batch from which the samples were selected. Generalising for n-sample homogenates, as n increases the resultant n-summed distribution $(P(x_t^n))$ tends towards a normal distribution shape coupled with an increase to the measures of location of the distribution (Fig 1 below). We can

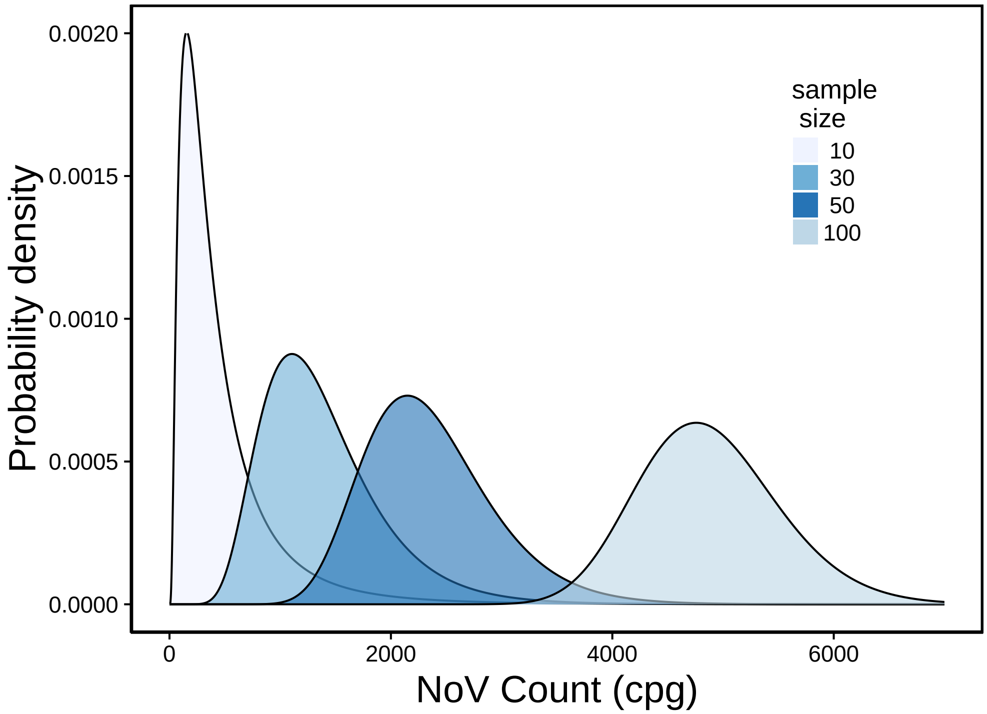

Fig 1. Shapes of n-summed distributions $P(x_{t,n})$. Distributions shown with sample sizes $n \in \{10, 30, 50, 100\}$

calculate parameters μ and σ to describe such n-summed lognormal distributions using the Fenton-Wilkinson approximation [2, 3]. This method shows that the distribution describing the sum of n independent and identically distributed random variables, where each $X_t^n \sim \mathcal{LN}(\mu_{X_t^n},\ \sigma^2_{X_t^n})$, have parameters $\sigma^2_{X_t^n} = \ln\left[1 + \left(\exp\left\{\sigma^2_{X_t}\right\} - 1\right)/n\right]$ and $\mu_{X_t^n} = \ln\left[n \exp\left\{\mu_{X_t}\right\}\right] + 0.5\left(\sigma^2_{X_t} - \sigma^2_{X_t^n}\right)$.

Applying these to the depuration distribution described in the main text as

$$P(x_t) = \frac{1}{\sqrt{2\pi}\sigma_0\ x_t} \exp\left\{\frac{-(\ln(x_t) + bt - \mu_0)^2}{2\sigma_0^2}\right\}, \qquad (1)$$

the parameters for a distribution of n-summed i.i.d. variables are defined as

$$\sigma_{t,n}^2 = \ln\left[1 + \frac{\left(\exp\left\{\sigma_t^2\right\} - 1\right)}{n}\right], \tag{2}$$

and

$$\begin{aligned} \mu_{t,n} &= \ln\left[n\exp\left\{\mu_t\right\}\right] + 0.5\left(\sigma_t^2 - \sigma_{t,n}^2\right) \\ \Rightarrow \mu_{t,n} &= \ln\left[n\exp\left\{\mu_0 - bt\right\}\right] + 0.5\left(\sigma_t^2 - \sigma_{t,n}^2\right). \end{aligned} \tag{3}$$

Eq 1, when used in conjunction with Eqs 2 and 3 above, allows us to construct density functions which describe the n-summed distribution of NoV (or *E.coli* or FRNA+) loads across a population of shellfish at any time t. This allows simulations of the current PCR testing procedure to be carried out for differing homogenate sizes by varying the value of n. PCR test simulations were carried for homogenate sample sizes $n \in \{1, 10, 20, 30, 40, 50, 60, 70, 80, 90, 100\}$, based on the lognormal distribution of NoV at $t = T_{WCV} = 225.9$ hrs. This is the MDT value shown in Table 4 of the main text, calculated from parameter values of $\bar{x}_0 = 1064$ cpg, $\Psi = 200$ cpg, $\varphi = 95\%$.

References

1. Lees D. International standardisation of a method for detection of human pathogenic viruses in molluscan shellfish. Food and Environmental Virology. 2010;2:146-155.

2. Cobb B, Salmer A. Approximating the Distribution of a Sum of Log-normal Random Variables. In: 6th European Workshop on Probabilistic Graphical Models, Granada, Spain, 2012.

3. Fenton L. The Sum of Log-Normal Probability Distributions in Scatter Transmission Systems. IRE Transactions on Communications Systems 1960;1:57–67.
