# Supplementary material for: A model for estimating pathogen variability in shellfish and predicting minimum depuration times
Source: PLoS One. 2018 Mar 7;13(3):e0193865. doi: 10.1371/journal.pone.0193865 (PMC5841822; doi:10.1371/journal.pone.0193865)
Supplement: S2 Fig — Results shown are based upon applying an assurance level φ = 95% (– – – line). (PDF) [file pone.0193865.s002.pdf]

S2 Text The following process was carried out to simulate testing of n-sized homogenates:

1. Construct $P(x_{t,n})$, the n-summed distribution as at time $t = T_{WCV}$;
2. Randomly select one variate $(x_{t,n})$ from $P(x_{t,n})$;
3. Divide the value of $x_{t,n}$ by n to adjust for the n-summation
4. Test against the value of Ψ where:

$$\begin{cases} \text{if } \frac{x_{t,n}}{n} < \Psi & \text{Pass} \\ \text{otherwise} & \text{Fail} \,. \end{cases}$$

For each $n \in \{1, 10, 20, 30, 40, 50, 60, 70, 80, 90, 100\}$, 30 sets of 1000 iterations of steps 1–4 were undertaken, and the results are shown in Fig 1.

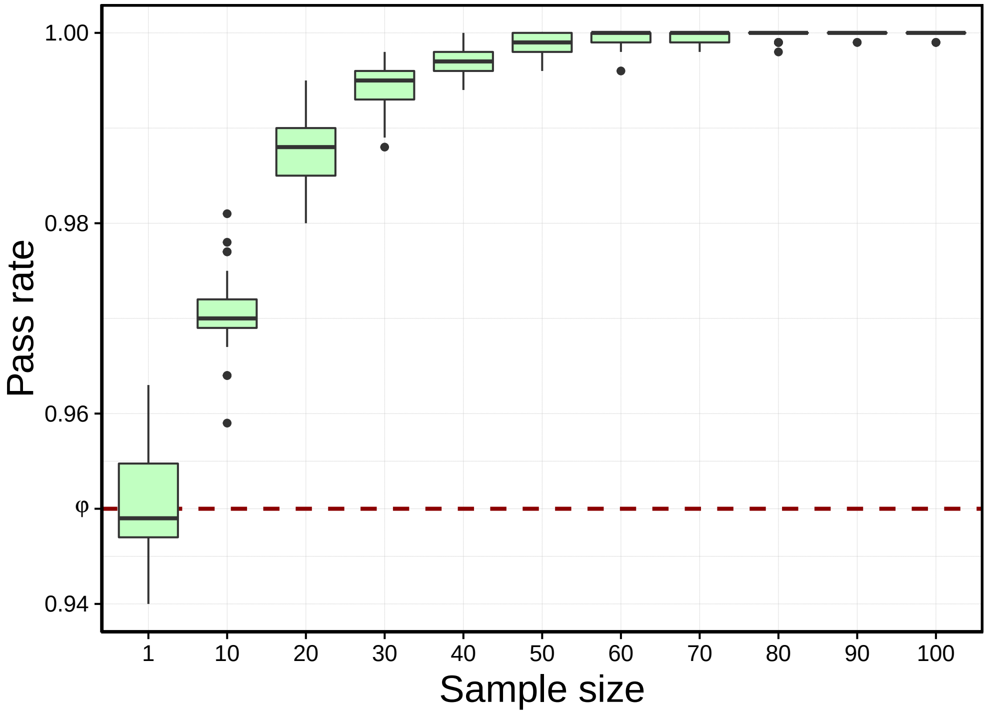

Fig 1. Results of test simulations of n-sized homogenates. Results shown are based upon applying an assurance level $\varphi = 95\%$ (− − − line)

Following on from Equation 3 in S1 Fig., we see that the mean contamination level (in cpg) decreases as sample size increases, as well as shifting the pathogen distribution towards a more normal (and less lognormal) shape. These result in an overall drop in the density of the tail of the distribution, and so the pass rate increases. The pass rates shown for the simulation using the current homogenate ($n = 10$) exhibit a range of 96% – 98%, encapsulating the pass rate for $\varphi = 95\%$ stated in main text (Table 3 - Minimum Depuration Times).
