# Supplementary material for: A model for estimating pathogen variability in shellfish and predicting minimum depuration times
Source: PLoS One. 2018 Mar 7;13(3):e0193865. doi: 10.1371/journal.pone.0193865 (PMC5841822; doi:10.1371/journal.pone.0193865)
Supplement: S3 Fig — Threshold limit Ψ = 200 NoV cpg, NoV assurance level φ = 95%, and initial mean NoV load x¯0=1064 cpg. (PDF) [file pone.0193865.s003.pdf]

S3 Text Figure 1 shows how minimum depuration time (MDT) follows a concave quadratic form with respect to variability (σ_0), as described in the main text by

$$T(\sigma_0) = b^{-1}\left[-\frac{1}{2}\sigma_0^2 + \sqrt{2}\mathrm{erf}^{-1}\left(2\varphi - 1\right)\sigma_0 + \ln\left(\frac{\bar{x}_0}{\Psi}\right)\right]. \quad (1)$$

The maximum MDT (or $\mathrm{MDT_{WCV}}$) occurs when $\sigma_0 = \sqrt{2}\mathrm{erf}^{-1}(2\varphi - 1)$, which is described as the worst case variability (WCV) with regards to MDT.

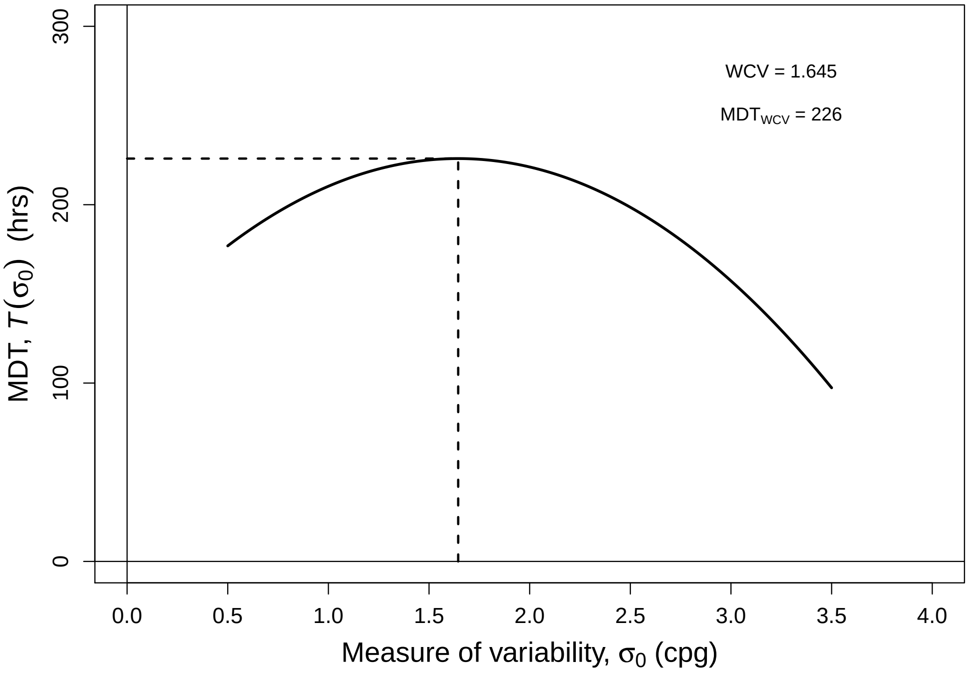

Fig 1. Plot of pathogen variability (σ_0) versus minimum depuration time ($T(\sigma_0)$). Threshold limit $\Psi = 200$ NoV cpg, NoV assurance level $\varphi = 95\%$, and initial mean NoV load $\bar{x}_0 = 1064$ cpg
